# Crystal structures of the solid solutions Na_3_Zn_0.912_Cd_0.088_B_5_O_10_ and Na_3_Zn_0.845_Mg_0.155_B_5_O_10_

**DOI:** 10.1107/S2056989017015249

**Published:** 2017-10-24

**Authors:** Xue-An Chen, Ya-Hua Zhang, Xin-An Chang, Wei-Qiang Xiao

**Affiliations:** a College of Materials Science and Engineering, Beijing University of Technology, Ping Le Yuan 100, Beijing 100124, People’s Republic of China; b Institute of Microstructure and Property of Advanced Materials, Beijing University of Technology, Ping Le Yuan 100, Beijing 100124, People’s Republic of China

**Keywords:** crystal structure, Na_3_ZnB_5_O_10_, solid solution, borate, isotypism

## Abstract

Two new penta­borates, tris­odium zinc cadmium penta­borate, Na_3_Zn_0.912_Cd_0.088_B_5_O_10_, and tris­odium zinc magnesium penta­borate, Na_3_Zn_0.845_Mg_0.155_B_5_O_10_, have been synthesized by high-temperature solution reactions at 1023 K. Their crystal structures were determined by single-crystal X-ray diffraction. Both solid solutions crystallize in the ortho­rhom­bic form of the parent compound Na_3_ZnB_5_O_10_ (space group type *Pbca*, *Z* = 8) and contain the double ring [B_5_O_10_]^5−^ anion composed of one BO_4_ tetra­hedron and four BO_3_ triangles as the basic structural motif. The anions are bridged by tetra­hedrally coordinated and occupationally disordered *M* ^2+^ (*M* = Zn/Cd, Zn/Mg) cations *via* common O atoms to form [*M*B_5_O_10_]_*n*_^3*n*−^ layers. The intra­layer inter­secting channels and the inter­layer voids are occupied by Na^+^ cations to balance the charge.

## Abstract 2

The solid solutions Na_3_Zn_0.912_Cd_0.088_B_5_O_10_ and Na_3_Zn_0.845_Mg_0.155_B_5_O_10_ adopt the ortho­rhom­bic form of the parent compound Na_3_ZnB_5_O_10_ where parts of the zinc cations are replaced by cadmium and magnesium cations, respectively.

## Chemical context   

Over the past few decades, borate materials have attracted increasing inter­est owing to their promising applications in non-linear optical materials, birefringent materials, ferroelectric and piezoelectric materials, and host materials for luminescence (Becker, 1998 ▸; Chen *et al.*, 1999 ▸). In general, boron atoms can be coordinated by either three or four oxygen atoms forming BO_3_ or BO_4_ groups, respectively. These groups may inter­connect with each other *via* common oxygen atoms to produce polyborate anionic groups that can adopt different coordination modes to bind to metal cations. The crystal chemistry of the resultant borates is rich, including infinite chains, sheets or networks for the anionic groups. For instance, in a series of penta­borates with general composition *A*_3_ *M*B_5_O_10_ (*A* = Na, K; *M* = Mg, Zn, Cd, Co, and Fe), at least three kinds of structure types have been reported, including K_2_NaZnB_5_O_10_ in space group *C*2/*c* (Chen *et al.*, 2010 ▸), *α*-Na_3_ZnB_5_O_10_, Na_3_CoB_5_O_10_ and K_3_ *M*B_5_O_10_ (*M* = Zn, Cd) in space group *P*2_1_ */n* (Chen *et al.*, 2007*a* ▸; Strauss *et al.*, 2016 ▸; Wu *et al.*, 2012 ▸; Yu *et al.*, 2011 ▸), and *β-*Na_3_ZnB_5_O_10_ as well as Na_3_ *M*B_5_O_10_ (*M* = Mg, Fe) in space group *Pbca* (Chen *et al.*, 2007*b* ▸, 2012 ▸; Strauss *et al.*, 2016 ▸). All of the structures contain polyborate anionic groups [B_5_O_10_]^5−^, which combine with different *A* ^+^ and *M* ^2+^ cations. During our exploratory syntheses of novel borate materials to study their structure–property relationships, we have obtained two new members of this family of compounds, *viz*. the solid solutions Na_3_Zn_0.912_Cd_0.088_B_5_O_10_ and Na_3_Zn_0.845_Mg_0.155_B_5_O_10_. Single crystal X-ray structure analyses revealed that these two compounds crystallize in the ortho­rhom­bic Na_3_ *M*B_5_O_10_ (*M* = Mg, Fe, Zn) structure type. Herein we describe their syntheses and crystal structures.

## Structural commentary   

**Figure 1:**
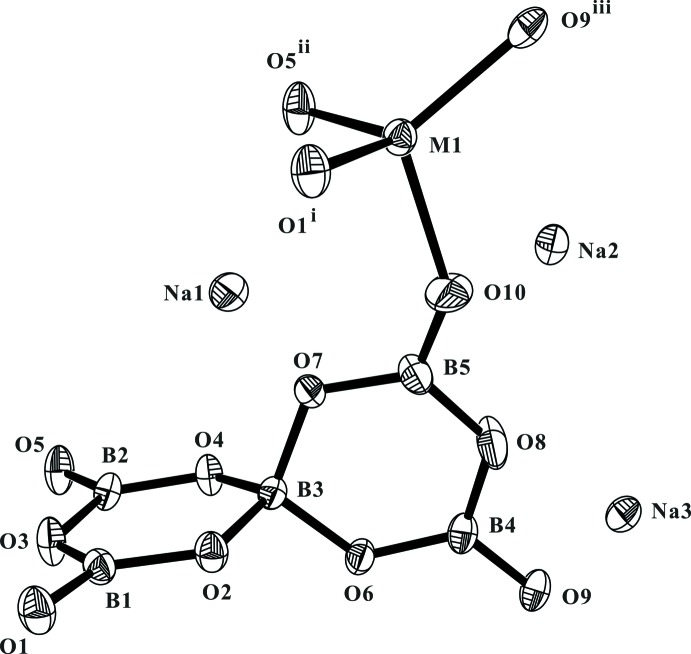
The asymmetric unit of Na_3_Zn_0.912_Cd_0.088_B_5_O_10_ supplemented by additional oxygen atoms to show the full coordination around the disordered *M* site (*M* = Zn_0.912 (4)_Cd_0.088 (4)_). Displacement ellipsoids are drawn at the 50% probability level. [Symmetry codes: (i) 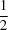 − *x*, 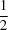 + *y*, *z*; (ii) 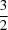 − *x*, 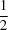 + *y*, *z*; (iii) 1 − *x*, 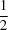 + *y*, 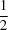 − *z*.]

**Figure 2:**
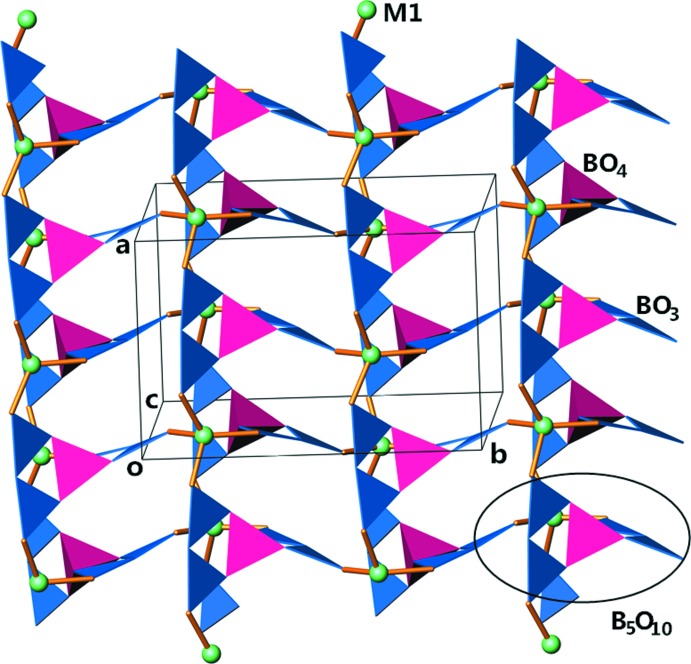
View of the [(Zn/Cd)B_5_O_10_]_*n*_^3*n*−^ layer approximately along [001]. (Zn/Cd) site: green spheres; BO_3_ groups: navy triangles; BO_4_ groups: magenta tetra­hedra.

**Figure 3:**
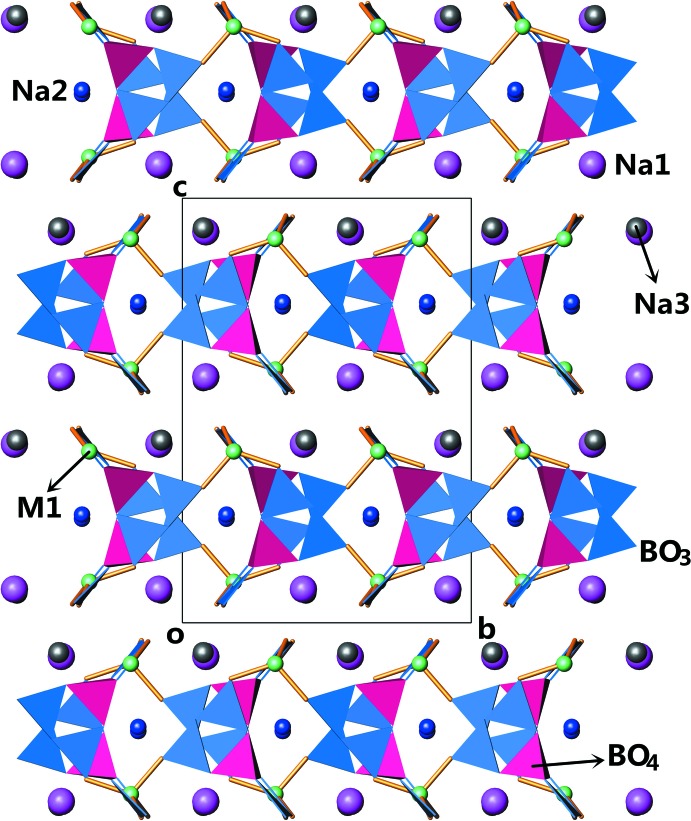
The crystal structure of Na_3_Zn_0.912_Cd_0.088_B_5_O_10_ projected along [100]. Na1 atoms: violet spheres; Na2 atoms: blue spheres; Na3 atoms: grey spheres; (Zn/Cd) atoms: green spheres; BO_3_ groups: navy triangles; BO_4_ groups: magenta tetra­hedra.

Since Na_3_Zn_0.912_Cd_0.088_B_5_O_10_ and Na_3_Zn_0.845_Mg_0.155_B_5_O_10_ have similar structures, the discussion will be based mainly on the cadmium-containing compound. The fundamental building blocks in this structure are [(Zn/Cd)O_4_] tetra­hedra and [B_5_O_10_]^5−^ groups, as illustrated in Fig. 1 ▸. Each [B_5_O_10_]^5−^ group has one BO_4_ tetra­hedron (T) and four BO_3_ triangles (Δ) condensed to a double ring *via* a common tetra­hedron, the connectivity of which can be formulated as 4Δ1T:<2ΔT> − <2ΔT> according to the nomenclature introduced by Burns *et al.* (1995 ▸). The penta­borate group comprises four terminal O atoms in its isolated form. Each [B_5_O_10_]^5−^ group is linked to four different [(Zn/Cd)O_4_] tetra­hedra and likewise each [(Zn/Cd)O_4_] tetra­hedron is connected to four neighbouring [B_5_O_10_]^5−^ groups through sharing all of the terminal O atoms, thus forming infinite sheets with an overall composition of [(Zn/Cd)B_5_O_10_]_*n*_^3*n*−^, as depicted in Fig. 2 ▸. The symmetry-equivalent (zinc/cadmium) borate sheets propagate in the *ab* plane and stack along the *c* axis. The sheets also afford inter­secting open channels running parallel to the *a-* and *b*-axis direc­tions. Fig. 3 ▸ shows a projection of the structure along [100]. Na2^+^ cations reside in these channels and Na1^+^ and Na3^+^ cations are situated at the voids between the sheets to provide charge compensation.

The asymmetric unit of Na_3_Zn_0.912_Cd_0.088_B_5_O_10_ comprises 19 independent sites, *i.e.* three Na, one disordered (Zn/Cd), five B, and ten O sites, all occupying general positions. Of the three unique Na sites, Na1 is surrounded by seven O atoms with Na—O distances divided into two sets: a set of five short ones is in the range 2.310 (3)–2.700 (3) Å, while another set includes two longer separations [3.054 (3)–3.059 (3) Å, Table 1 ▸]. Bond-valence-sum (BVS) calculations using Brown’s formula (Brown & Altermatt, 1985 ▸) gave a BVS value of 0.89 valence units (v.u.) for the seven-coordinated Na1 cation, confirming that the long bonds participate in the overall metal coordination sphere. The coordination environment can be described as an irregular polyhedron. Similarly, Na2 and Na3 atoms have also adopted the seven-coordinated irregular polyhedral arrangement. This is different from the situation in monoclinic *α-*Na_3_ZnB_5_O_10_, where three distinct Na sites have coordination numbers of six, seven, and eight, respectively (Chen *et al.*, 2007*a* ▸). In the Na_3_Zn_0.912_Cd_0.088_B_5_O_10_ structure, the Na—O distances fall in the range 2.273 (3)–3.059 (3) Å (average range for the three sites 2.553–2.657 Å), which is similar to the value reported for the seven-coordinated Na^+^ cation in *α-*Na_3_ZnB_5_O_10_ [2.318 (2)–2.859 (3) Å, average 2.531 Å] (Chen *et al.*, 2007*a* ▸), and in agreement with the value of 2.50 Å computed from crystal radii sums for seven-coord­inated Na^+^ and four-coordinated O^2−^ ions (Shannon, 1976 ▸). In the Na_3_Zn_0.912_Cd_0.088_B_5_O_10_ structure, the *M*1 site is statistically disordered with Zn^2+^ and Cd^2+^ cations. The [Zn_0.912 (4)_Cd_0.088 (4)_O_4_] tetra­hedron exhibits a mean O—*M*1—O angle of 109.14°, close to the ideal value of 109.5°. *M*1—O bond lengths [1.974 (2)–2.000 (2) Å] are normal when compared with those observed in the related structures of CdZn_2_(BO_3_)_2_ [(Zn_0.67_Cd_0.33_)–O = 1.995 (14)–2.130 (15) Å, CN = 4] (Zhang *et al.*, 2008 ▸), Cd_3_Zn_3_(BO_3_)_4_ [(Zn_0.5_Cd_0.5_)–O = 2.015 (3)–2.131 (4) Å, CN = 4] (Sun *et al.*, 2003 ▸), and Cd_1.17_Zn_0.83_B_2_O_5_ [(Zn_0.753_Cd_0.247_)–O = 1.997 (7)–2.109 (6) Å, CN = 4] (Yuan *et al.*, 2005 ▸). Of the boron sites, B3 has a tetra­hedral configuration, while other B sites are in triangular configurations. The BO_4_ and BO_3_ groups are rather regular, with average O—B—O angles being close to 109.5 or 120°, respectively. The B—O bond lengths in the tetra­hedron cover the range between 1.467 (4) and 1.472 (4) Å, and those in the triangles between 1.305 (5) and 1.407 (4) Å. The average B—O bond lengths (1.469 Å and 1.366–1.373 Å, respectively) are in good agreement with the data reviewed by Hawthorne *et al.* (1996 ▸). The calculated BVS values concerning B atoms are around 3 v.u., ranging from 2.99 v.u. for B1 to 3.07 v.u. for B3.

A comparison between the Na_3_Zn_0.845_Mg_0.155_B_5_O_10_ and Na_3_Zn_0.912_Cd_0.088_B_5_O_10_ structures reveals that the isovalent substitution of Mg^2+^ for Cd^2+^ ions in Na_3_Zn_0.912_Cd_0.088_B_5_O_10_ leads to a significant decrease in the cell volume [*V* = 1749.7 (3) Å^3^ for the (Zn/Mg) *vs* 1763.7 (5) Å^3^ for the (Zn/Cd) phase; *V* = 1745.50 (17) Å^3^ for unsubstituted Na_3_ZnB_5_O_10_ (Chen *et al.*, 2012 ▸)]. In the two solid solutions, the [B_5_O_10_]^5−^ groups show a similar configuration, with the dihedral angles between two hexa­gonal ring planes being identical within the experimental error [84.7 (1) *vs* 84.9 (1)°]. The geometric parameters of BO_3_ triangles and BO_4_ tetra­hedra remain basically unchanged from the (Zn/Cd) to the (Zn/Mg) phase, while the [NaO_7_] polyhedra in the (Zn/Mg) compound are slightly smaller compared with the corresponding ones in the (Zn/Cd) compound. In contrast, a remarkable difference in the coordination geometry around the divalent metal ions exists. The average (Zn/Mg)—O bond length is 1.962 Å, shorter than the average (Zn/Cd)—O bond length of 1.990 Å. The O—(Zn/Mg)—O angles are 98.11 (13)–119.58 (11)°, distinctly narrower than the O—(Zn/Cd)—O angles of 96.64 (12)–120.23 (10)°. The [(Zn/Mg)O_4_] tetra­hedron appears to be smaller and more regular than the [(Zn/Cd)O_4_] tetra­hedron, which follows the general trend of the respective ionic radii [*r*(Mg^2+^) = 0.72 < *r*(Zn^2+^) = 0.74 < *r*(Cd^2+^) = 0.92 Å, CN = 4] (Shannon, 1976 ▸).

As mentioned above, Na_3_ZnB_5_O_10_ was reported to exist in two structural variants, which we name in the following the *α*- and *β-*phases, respectively. The *α*-form crystallizes in space group *P*2_1_ */n*, while the *β-*form in space group *Pbca* (Chen *et al.*, 2007*a* ▸, 2012 ▸). The relationship between their crystal structures follows a group–subgroup relation: *β-*Na_3_ZnB_5_O_10_ (*Pbca*, **a**, **b**, **c**, *Z* = 8) → *α*-Na_3_ZnB_5_O_10_ (*P*2_1_ */n*, which is a maximal non-isomorphic subgroup of index 2 of *Pbca*, 0.5**a** + 0.5**b, c**, 0.5**a** − 0.5**b**, *Z* = 4). *β-*Na_3_ZnB_5_O_10_ is isotypic with Na_3_ *M*B_5_O_10_ (*M* = Mg, Fe), and the title compounds which are substitutional solid solutions of *β-*Na_3_ZnB_5_O_10_. All structures comprise identical [*M*B_5_O_10_]_*n*_^3*n*−^ (*M* = Zn, Mg, Fe, (Zn/Mg), (Zn/Cd)) layers constructed by [B_5_O_10_]^5−^ groups and *M*O_4_ tetra­hedra *via* common O atoms, and the coordin­ation environments around all cationic sites are very similar. The main differences pertain to the [*M*O_4_] tetra­hedra. For example, the average *M*—O bond lengths are 1.963, 1.963, 1.962, and 1.990 Å for *β-*Na_3_ZnB_5_O_10_, Na_3_MgB_5_O_10_, Na_3_Zn_0.845_Mg_0.155_B_5_O_10_, and Na_3_Zn_0.912_Cd_0.088_B_5_O_10_, respectively. The cell volumes show a similar trend.

For Na_3_ZnB_5_O_10_, the present study indicates that a partial replacement of Zn^2+^ by Cd^2+^ or Mg^2+^ is favourable for the formation of the ortho­rhom­bic *Pbca* phase. However, keeping the Na^+^ ions unchanged, the complete replacement of Zn^2+^ by larger Cd^2+^ ions does not result in the isotypic cadmium analogue. We have attempted to prepare a hypothetical compound with nominal composition ‘Na_3_CdB_5_O_10_’ *via* a standard solid-state synthetic route by mixing stoichiometric amounts of Na_2_CO_3_, CdO, and H_3_BO_3_ powders followed by annealing the mixture at a temperature of 873 K in air for several weeks. No ‘Na_3_CdB_5_O_10_’ has been obtained, only a mixture of known phases, *viz.* NaBO_2_ and Cd_2_B_2_O_5_, was formed instead, according to powder X-ray diffraction analyses. This indicates that the structural variants in the family of compounds *A*_3_ *M*B_5_O_10_ depend strongly on sizes of *A* ^+^ and *M* ^2+^ cations.

## Synthesis and crystallization   

In a typical synthesis of the cadmium-containing compound, a powder mixture of the starting materials Na_2_B_4_O_7_·10H_2_O, ZnO, CdO, H_3_BO_3_ in the molar ratio Na:Zn:Cd:B = 3:2:1:7 was transferred to a platinum crucible of 40 mm in diameter and 40 mm in height. The sample was melted at 1023 K for one week, then cooled down to 773 K at a rate of 0.5 K h^−1^, to 573 K at 1.0 K h^−1^, followed by cooling to room temperature at 20 K h^−1^. Colourless prismatic crystals were isolated from the solidified melt. Energy-dispersive X-ray analyses (EDX) in a scanning electron microscope confirmed the existence of the heavy elements zinc and cadmium with an approximate atomic ratio of 8.2:1.5, close to the refined composition of the crystal (9.12: 0.88) (see Figs. S1–S2 and Table S1 in the *Supporting information*). The magnesium-containing compound was prepared in the same way, except that the starting materials were Na_2_B_4_O_7_·10H_2_O, ZnO, MgO, H_3_BO_3_ in the molar ratio Na:Zn:Mg:B = 2:2:1:6. EDX measurements for the Na_3_Zn_0.845_Mg_0.155_B_5_O_10_ crystal gave an approximate atomic ratio of Zn:Mg = 4.9:3.8, deviating significantly from the refined composition (8.45:1.55) (see Figs. S3–S4 and Table S2). This may be due to the fact that the Mg-peak in the EDX spectrum is very close to the main peak of Zn, which leads the calculations of the integrated intensities of the Zn and Mg peaks to be inaccurate, consequently producing an inaccurate Zn/Mg atomic ratio. The powder X-ray diffraction pattern of the ground crystals are in good agreement with those calculated from the single-crystal data.

The infrared spectra exhibit the characteristic absorption bands of both BO_3_ and BO_4_ groups for Na_3_Zn_0.912_Cd_0.088_B_5_O_10_ (Na_3_Zn_0.845_Mg_0.155_B_5_O_10_), *i.e*. BO_3_ asymmetric stretching vibrations in the frequency range 1400–1206 (1400–1201) cm^−1^, BO_4_ asymmetric stretching modes from 1077 to 1025 (1079 to 1026) cm^−1^, BO_3_ symmetric stretching modes lying at around 938 (939) cm^−1^, BO_4_ symmetric stretching mixed with BO_3_ out-of plane bending modes locating at about 776 (777) cm^−1^, and the overlapped BO_3_ and BO_4_ bending vibrations occurring below 722 (723) cm^−1^. These values correspond well to those reported in the literature (Filatov *et al.*, 2004 ▸). UV–VIS diffuse reflectance spectra indicated insulator character, with optical band gaps of about 2.95 and 3.10 eV for Na_3_Zn_0.912_Cd_0.088_B_5_O_10_ and Na_3_Zn_0.845_Mg_0.155_B_5_O_10_, respectively.

## Refinement   

Crystal data, data collection and structure refinement details are summarized in Table 2 ▸. Based on the EDX measurements, cadmium and magnesium, respectively, was incorporated in the crystals. In fact, refinements of the occupancies of the zinc sites in the two structures revealed a small incorporation of cadmium and a somewhat higher incorporation of magnesium, respectively. For the final models, the occupancies of the disordered *M* sites (*M* = Zn, Cd and Zn, Mg, respectively) were constrained to 1.0, with the same coordinates and displacement parameters for the two types of metals. The refined ratios were Zn_0.912 (4)_:Cd_0.088 (4)_ and Zn_0.845 (5)_:Mg_0.155 (5)_, respectively. The largest residual electron densities in the final difference-Fourier map are below 1.59 e Å^−3^.

## Supplementary Material

Crystal structure: contains datablock(s) I, II, global. DOI: 10.1107/S2056989017015249/wm5422sup1.cif

Structure factors: contains datablock(s) I. DOI: 10.1107/S2056989017015249/wm5422Isup2.hkl

Structure factors: contains datablock(s) II. DOI: 10.1107/S2056989017015249/wm5422IIsup3.hkl

CCDC references: 1580783, 1580782

Additional supporting information:  crystallographic information; 3D view; checkCIF report

## Figures and Tables

**Table 1:** Selected geometric parameters (Å, °) for (I)

Na1—O1^i^	2.310 (3)	Zn1—O5^iii^	1.974 (2)
Na1—O9^ii^	2.404 (3)	Zn1—O9^iv^	1.985 (2)
Na1—O4	2.487 (3)	Zn1—O10	2.000 (2)
Na1—O7	2.587 (3)	Zn1—O1^vi^	2.000 (2)
Na1—O3^iii^	2.700 (3)	B1—O1	1.335 (4)
Na1—O3^i^	3.054 (3)	B1—O2	1.384 (4)
Na1—O8^ii^	3.059 (3)	B1—O3	1.400 (4)
Na2—O2^iv^	2.355 (3)	B2—O5	1.324 (4)
Na2—O6^iii^	2.381 (3)	B2—O4	1.376 (4)
Na2—O10^ii^	2.388 (3)	B2—O3	1.407 (4)
Na2—O6^iv^	2.550 (3)	B3—O4	1.467 (4)
Na2—O4^iii^	2.618 (3)	B3—O2	1.468 (4)
Na2—O10	2.719 (3)	B3—O7	1.468 (4)
Na2—O8	2.859 (3)	B3—O6	1.472 (4)
Na3—O5^v^	2.273 (3)	B4—O9	1.337 (4)
Na3—O9	2.361 (3)	B4—O6	1.369 (4)
Na3—O7^ii^	2.438 (3)	B4—O8	1.395 (4)
Na3—O1^iv^	2.458 (3)	B5—O10	1.305 (5)
Na3—O3^v^	2.786 (3)	B5—O7	1.388 (4)
Na3—O10^ii^	2.868 (3)	B5—O8	1.405 (5)
Na3—O2^ii^	2.910 (3)		
			
O5^iii^—Zn1—O9^iv^	111.39 (10)	O4—B3—O2	110.6 (3)
O5^iii^—Zn1—O10	103.73 (11)	O4—B3—O7	109.8 (3)
O9^iv^—Zn1—O10	112.42 (10)	O2—B3—O7	109.4 (3)
O5^iii^—Zn1—O1^vi^	110.40 (9)	O4—B3—O6	107.9 (3)
O9^iv^—Zn1—O1^vi^	120.23 (10)	O2—B3—O6	108.8 (3)
O10—Zn1—O1^vi^	96.64 (12)	O7—B3—O6	110.4 (3)
O1—B1—O2	121.5 (3)	O9—B4—O6	123.6 (3)
O1—B1—O3	120.0 (3)	O9—B4—O8	118.8 (3)
O2—B1—O3	118.4 (3)	O6—B4—O8	117.6 (3)
O5—B2—O4	123.4 (3)	O10—B5—O7	123.2 (3)
O5—B2—O3	117.4 (3)	O10—B5—O8	118.1 (3)
O4—B2—O3	119.1 (3)	O7—B5—O8	118.7 (3)

Symmetry codes: (i) 
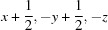
; (ii) 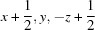; (iii) 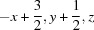; (iv) 
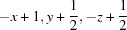
; (v) 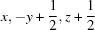; (vi) 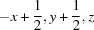.

**Table 2:** Experimental details

	Na_3_Zn_0.912_Cd_0.088_B_5_O_10_	Na_3_Zn_0.845_Mg_0.155_B_5_O_10_
Crystal data		
*M*_r_	352.53	342.03
Crystal system, space group	Orthorhombic, *P* *b* *c* *a*	Orthorhombic, *P* *b* *c* *a*
Temperature (K)	293	293
*a*, *b*, *c* (Å)	7.9407 (14), 12.293 (2), 18.0684 (19)	7.8931 (12), 12.2555 (12), 18.0874 (11)
*V* (Å^3^)	1763.7 (5)	1749.7 (3)
*Z*	8	8
Radiation type	Mo *K*α	Mo *K*α
μ (mm^−1^)	2.95	2.60
Crystal size (mm)	0.30 × 0.10 × 0.10	0.30 × 0.20 × 0.20

Data collection		
Diffractometer	Rigaku AFC-7R	Rigaku AFC-7R
Absorption correction	ψ scan (Kopfmann & Huber, 1968 ▸)	ψ scan (Kopfmann & Huber, 1968 ▸)
*T*_min_, *T*_max_	0.703, 0.752	0.532, 0.603
No. of measured, independent and observed [*I* > 2σ(*I*)] reflections	3006, 2562, 1557	2982, 2542, 1496
*R*_int_	0.061	0.053
(sin θ/λ)_max_ (Å^−1^)	0.703	0.703

Refinement		
*R*[*F* ^2^ > 2σ(*F* ^2^)], *wR*(*F* ^2^), *S*	0.037, 0.072, 0.91	0.044, 0.113, 0.87
No. of reflections	2562	2542
No. of parameters	174	173
Δρ_max_, Δρ_min_ (e Å^−3^)	0.45, −0.45	1.59, −0.70

Computer programs: *Rigaku/AFC Diffractometer Control Software* (Rigaku Corporation, 1994 ▸), *SHELXS97* (Sheldrick, 2008 ▸), *SHELXL2014* (Sheldrick, 2015 ▸), *ATOMS* (Dowty, 1999 ▸) and *publCIF* (Westrip, 2010 ▸).

## supplementary crystallographic information

### Trisodium zinc cadmium pentaborate (I) Crystal data

**Table:**

Na_3_Zn_0.912_Cd_0.088_B_5_O_10_	*D*_x_ = 2.655 Mg m^−^^3^
*M**_r_* = 352.53	Mo *K*α radiation, λ = 0.71073 Å
Orthorhombic, *P**b**c**a*	Cell parameters from 25 reflections
*a* = 7.9407 (14) Å	θ = 12.1–22.2°
*b* = 12.293 (2) Å	µ = 2.95 mm^−^^1^
*c* = 18.0684 (19) Å	*T* = 293 K
*V* = 1763.7 (5) Å^3^	Prism, colorless
*Z* = 8	0.30 × 0.10 × 0.10 mm
*F*(000) = 1356.7	

### Trisodium zinc cadmium pentaborate (I) Data collection

**Table:**

Rigaku AFC-7R diffractometer	*R*_int_ = 0.061
Radiation source: fine-focus sealed tube	θ_max_ = 30.0°, θ_min_ = 2.3°
2θ – ω scans	*h* = 0→11
Absorption correction: ψ scan (Kopfmann & Huber, 1968)	*k* = 0→17
*T*_min_ = 0.703, *T*_max_ = 0.752	*l* = 0→25
3006 measured reflections	3 standard reflections every 150 reflections
2562 independent reflections	intensity decay: 2.2%
1557 reflections with *I* > 2σ(*I*)	

### Trisodium zinc cadmium pentaborate (I) Refinement

**Table:**

Refinement on *F*^2^	0 restraints
Least-squares matrix: full	*w* = 1/[σ^2^(*F*_o_^2^) + (0.0224*P*)^2^] where *P* = (*F*_o_^2^ + 2*F*_c_^2^)/3
*R*[*F*^2^ > 2σ(*F*^2^)] = 0.037	(Δ/σ)_max_ = 0.001
*wR*(*F*^2^) = 0.072	Δρ_max_ = 0.45 e Å^−^^3^
*S* = 0.91	Δρ_min_ = −0.45 e Å^−^^3^
2562 reflections	Extinction correction: SHELXL2014 (Sheldrick, 2015), Fc^*^=kFc[1+0.001xFc^2^λ^3^/sin(2θ)]^-1/4^
174 parameters	Extinction coefficient: 0.00127 (16)

### Trisodium zinc cadmium pentaborate (I) Special details

**Table:**

Geometry. All esds (except the esd in the dihedral angle between two l.s. planes) are estimated using the full covariance matrix. The cell esds are taken into account individually in the estimation of esds in distances, angles and torsion angles; correlations between esds in cell parameters are only used when they are defined by crystal symmetry. An approximate (isotropic) treatment of cell esds is used for estimating esds involving l.s. planes.

### Trisodium zinc cadmium pentaborate (I) Fractional atomic coordinates and isotropic or equivalent isotropic displacement parameters (Å^2^)

**Table:**

	*x*	*y*	*z*	*U*_iso_*/*U*_eq_	Occ. (<1)
Na1	0.8291 (2)	0.41887 (12)	0.07922 (8)	0.0318 (4)	
Na2	0.69268 (17)	0.65268 (11)	0.25478 (7)	0.0248 (3)	
Na3	0.79023 (18)	0.42762 (12)	0.42997 (7)	0.0271 (4)	
Zn1	0.42413 (4)	0.67826 (3)	0.09632 (2)	0.01812 (12)	0.912 (4)
Cd1	0.42413 (4)	0.67826 (3)	0.09632 (2)	0.01812 (12)	0.088 (4)
B1	0.4192 (5)	0.1571 (3)	0.0744 (2)	0.0195 (8)	
B2	0.7256 (5)	0.1729 (4)	0.07257 (19)	0.0198 (8)	
B3	0.5612 (5)	0.2868 (3)	0.16320 (19)	0.0174 (7)	
B4	0.5534 (5)	0.3497 (3)	0.2967 (2)	0.0206 (8)	
B5	0.4884 (6)	0.4782 (3)	0.1978 (2)	0.0239 (9)	
O1	0.2779 (3)	0.1189 (2)	0.04400 (13)	0.0260 (6)	
O2	0.4154 (3)	0.22647 (18)	0.13473 (12)	0.0211 (5)	
O3	0.5761 (3)	0.1244 (2)	0.04691 (13)	0.0261 (6)	
O4	0.7176 (3)	0.2455 (2)	0.13046 (12)	0.0215 (5)	
O5	0.8686 (3)	0.1418 (2)	0.04139 (13)	0.0286 (6)	
O6	0.5712 (3)	0.27092 (17)	0.24380 (12)	0.0220 (5)	
O7	0.5416 (3)	0.40273 (18)	0.14568 (12)	0.0226 (6)	
O8	0.5100 (4)	0.4541 (2)	0.27321 (13)	0.0380 (8)	
O9	0.5732 (3)	0.33154 (19)	0.36916 (11)	0.0241 (5)	
O10	0.4228 (4)	0.5721 (2)	0.18019 (14)	0.0390 (7)	

### Trisodium zinc cadmium pentaborate (I) Atomic displacement parameters (Å^2^)

**Table:**

	*U*^11^	*U*^22^	*U*^33^	*U*^12^	*U*^13^	*U*^23^
Na1	0.0399 (9)	0.0298 (8)	0.0258 (8)	−0.0017 (7)	−0.0048 (7)	0.0019 (6)
Na2	0.0245 (7)	0.0292 (7)	0.0206 (6)	−0.0011 (7)	0.0013 (6)	−0.0035 (7)
Na3	0.0333 (8)	0.0298 (8)	0.0184 (6)	0.0006 (7)	−0.0012 (6)	0.0029 (6)
Zn1	0.01764 (18)	0.01970 (19)	0.01702 (17)	−0.00189 (18)	0.00014 (17)	−0.00138 (16)
Cd1	0.01764 (18)	0.01970 (19)	0.01702 (17)	−0.00189 (18)	0.00014 (17)	−0.00138 (16)
B1	0.0197 (17)	0.0201 (19)	0.0188 (16)	−0.0030 (19)	−0.0011 (17)	−0.0027 (14)
B2	0.0202 (18)	0.026 (2)	0.0135 (15)	0.0030 (19)	−0.0009 (14)	−0.0027 (17)
B3	0.0195 (18)	0.0194 (16)	0.0134 (15)	−0.0001 (16)	−0.0001 (16)	−0.0009 (13)
B4	0.0191 (19)	0.027 (2)	0.0160 (17)	0.0037 (17)	−0.0023 (15)	−0.0037 (15)
B5	0.028 (2)	0.019 (2)	0.025 (2)	0.0007 (17)	−0.0021 (18)	−0.0050 (17)
O1	0.0208 (13)	0.0307 (14)	0.0266 (13)	−0.0029 (12)	−0.0025 (12)	−0.0080 (11)
O2	0.0178 (11)	0.0250 (12)	0.0204 (11)	−0.0016 (11)	−0.0011 (11)	−0.0049 (9)
O3	0.0212 (12)	0.0332 (14)	0.0239 (12)	0.0000 (13)	0.0006 (12)	−0.0142 (11)
O4	0.0187 (12)	0.0255 (13)	0.0204 (11)	0.0009 (11)	−0.0005 (10)	−0.0092 (11)
O5	0.0214 (12)	0.0390 (15)	0.0253 (13)	0.0061 (12)	0.0001 (11)	−0.0140 (12)
O6	0.0318 (13)	0.0200 (11)	0.0142 (10)	0.0056 (12)	0.0008 (11)	−0.0002 (9)
O7	0.0358 (16)	0.0149 (11)	0.0173 (11)	0.0007 (11)	−0.0015 (10)	−0.0009 (9)
O8	0.068 (2)	0.0257 (14)	0.0206 (13)	0.0200 (14)	−0.0123 (14)	−0.0084 (11)
O9	0.0271 (12)	0.0320 (13)	0.0133 (10)	0.0012 (14)	−0.0030 (11)	−0.0030 (10)
O10	0.061 (2)	0.0242 (13)	0.0322 (14)	0.0203 (15)	0.0125 (15)	0.0114 (11)

### Trisodium zinc cadmium pentaborate (I) Geometric parameters (Å, º)

**Table:**

Na1—O1^i^	2.310 (3)	Zn1—Na1^iii^	3.5615 (16)
Na1—O9^ii^	2.404 (3)	B1—O1	1.335 (4)
Na1—O4	2.487 (3)	B1—O2	1.384 (4)
Na1—O7	2.587 (3)	B1—O3	1.400 (4)
Na1—O3^iii^	2.700 (3)	B1—Cd1^xi^	2.767 (4)
Na1—B4^ii^	2.986 (4)	B1—Na1^xii^	3.015 (4)
Na1—B1^i^	3.015 (4)	B2—O5	1.324 (4)
Na1—O3^i^	3.054 (3)	B2—O4	1.376 (4)
Na1—O8^ii^	3.059 (3)	B2—O3	1.407 (4)
Na1—B3	3.076 (4)	B2—Na3^xiii^	2.903 (4)
Na1—B2	3.135 (5)	B2—Na1^v^	3.155 (5)
Na1—B2^iii^	3.155 (5)	B3—O4	1.467 (4)
Na1—Na3^iv^	3.4250 (19)	B3—O2	1.468 (4)
Na1—Cd1^v^	3.5614 (16)	B3—O7	1.468 (4)
Na2—O2^vi^	2.355 (3)	B3—O6	1.472 (4)
Na2—O6^iii^	2.381 (3)	B3—Na2^xiv^	2.996 (4)
Na2—O10^ii^	2.388 (3)	B3—Na2^v^	3.046 (4)
Na2—O6^vi^	2.550 (3)	B4—O9	1.337 (4)
Na2—O4^iii^	2.618 (3)	B4—O6	1.369 (4)
Na2—O10	2.719 (3)	B4—O8	1.395 (4)
Na2—O8	2.859 (3)	B4—Na1^x^	2.986 (4)
Na2—B5	2.879 (4)	B5—O10	1.305 (5)
Na2—B3^vi^	2.996 (4)	B5—O7	1.388 (4)
Na2—B3^iii^	3.046 (4)	B5—O8	1.405 (5)
Na2—Zn1^ii^	3.2734 (14)	B5—Na3^x^	2.862 (4)
Na2—Cd1^ii^	3.2734 (14)	O1—Cd1^xi^	2.000 (2)
Na3—O5^vii^	2.273 (3)	O1—Zn1^xi^	2.000 (2)
Na3—O9	2.361 (3)	O1—Na1^xii^	2.310 (3)
Na3—O7^ii^	2.438 (3)	O1—Na3^xiv^	2.458 (3)
Na3—O1^vi^	2.458 (3)	O2—Na2^xiv^	2.355 (3)
Na3—O3^vii^	2.786 (3)	O2—Na3^x^	2.910 (3)
Na3—B5^ii^	2.862 (4)	O3—Na1^v^	2.700 (3)
Na3—O10^ii^	2.868 (3)	O3—Na3^xiii^	2.786 (3)
Na3—B2^vii^	2.903 (4)	O4—Na2^v^	2.618 (3)
Na3—O2^ii^	2.910 (3)	O5—Cd1^v^	1.974 (2)
Na3—Cd1^ii^	3.2938 (16)	O5—Zn1^v^	1.974 (2)
Na3—Zn1^ii^	3.2938 (16)	O5—Na3^xiii^	2.273 (3)
Na3—Na1^viii^	3.4250 (19)	O6—Na2^v^	2.381 (3)
Zn1—O5^iii^	1.974 (2)	O6—Na2^xiv^	2.550 (3)
Zn1—O9^vi^	1.985 (2)	O7—Na3^x^	2.438 (3)
Zn1—O10	2.000 (2)	O9—Cd1^xiv^	1.985 (2)
Zn1—O1^ix^	2.000 (2)	O9—Zn1^xiv^	1.985 (2)
Zn1—Na2^x^	3.2733 (14)	O9—Na1^x^	2.404 (3)
Zn1—Na3^x^	3.2938 (16)	O10—Na2^x^	2.388 (3)
Zn1—Na3^vi^	3.5382 (16)	O10—Na3^x^	2.868 (3)
			
O1^i^—Na1—O9^ii^	115.21 (10)	O10^ii^—Na3—Zn1^ii^	37.07 (5)
O1^i^—Na1—O4	97.12 (10)	B2^vii^—Na3—Zn1^ii^	125.68 (9)
O9^ii^—Na1—O4	76.10 (9)	O2^ii^—Na3—Zn1^ii^	128.80 (7)
O1^i^—Na1—O7	106.07 (10)	Cd1^ii^—Na3—Zn1^ii^	0.000 (13)
O9^ii^—Na1—O7	119.81 (9)	O5^vii^—Na3—Na1^viii^	65.50 (8)
O4—Na1—O7	56.45 (8)	O9—Na3—Na1^viii^	116.11 (8)
O1^i^—Na1—O3^iii^	91.64 (9)	O7^ii^—Na3—Na1^viii^	137.47 (8)
O9^ii^—Na1—O3^iii^	106.08 (10)	O1^vi^—Na3—Na1^viii^	42.40 (6)
O4—Na1—O3^iii^	169.04 (9)	O3^vii^—Na3—Na1^viii^	50.25 (6)
O7—Na1—O3^iii^	114.72 (9)	B5^ii^—Na3—Na1^viii^	131.86 (10)
O1^i^—Na1—B4^ii^	140.44 (12)	O10^ii^—Na3—Na1^viii^	107.87 (7)
O9^ii^—Na1—B4^ii^	25.95 (9)	B2^vii^—Na3—Na1^viii^	59.13 (9)
O4—Na1—B4^ii^	71.88 (10)	O2^ii^—Na3—Na1^viii^	151.51 (7)
O7—Na1—B4^ii^	98.98 (10)	Cd1^ii^—Na3—Na1^viii^	71.79 (4)
O3^iii^—Na1—B4^ii^	105.21 (10)	Zn1^ii^—Na3—Na1^viii^	71.79 (4)
O1^i^—Na1—B1^i^	24.80 (10)	O5^iii^—Zn1—O9^vi^	111.39 (10)
O9^ii^—Na1—B1^i^	91.57 (10)	O5^iii^—Zn1—O10	103.73 (11)
O4—Na1—B1^i^	99.30 (10)	O9^vi^—Zn1—O10	112.42 (10)
O7—Na1—B1^i^	127.81 (11)	O5^iii^—Zn1—O1^ix^	110.40 (9)
O3^iii^—Na1—B1^i^	91.42 (10)	O9^vi^—Zn1—O1^ix^	120.23 (10)
B4^ii^—Na1—B1^i^	117.46 (12)	O10—Zn1—O1^ix^	96.64 (12)
O1^i^—Na1—B3	104.34 (11)	O5^iii^—Zn1—Na2^x^	149.29 (8)
O9^ii^—Na1—B3	97.50 (10)	O9^vi^—Zn1—Na2^x^	80.73 (7)
O4—Na1—B3	28.10 (9)	O10—Zn1—Na2^x^	46.49 (8)
O7—Na1—B3	28.40 (9)	O1^ix^—Zn1—Na2^x^	84.43 (7)
O3^iii^—Na1—B3	142.49 (10)	O5^iii^—Zn1—Na3^x^	89.10 (8)
B4^ii^—Na1—B3	83.80 (11)	O9^vi^—Zn1—Na3^x^	159.51 (8)
B1^i^—Na1—B3	117.05 (11)	O10—Zn1—Na3^x^	59.81 (9)
O1^i^—Na1—B2	73.94 (10)	O1^ix^—Zn1—Na3^x^	48.05 (8)
O9^ii^—Na1—B2	78.20 (10)	Na2^x^—Zn1—Na3^x^	81.22 (3)
O4—Na1—B2	25.10 (8)	O5^iii^—Zn1—Na3^vi^	122.04 (8)
O7—Na1—B2	73.29 (9)	O9^vi^—Zn1—Na3^vi^	39.19 (7)
O3^iii^—Na1—B2	165.25 (10)	O10—Zn1—Na3^vi^	131.64 (8)
B4^ii^—Na1—B2	84.87 (11)	O1^ix^—Zn1—Na3^vi^	82.34 (8)
B1^i^—Na1—B2	74.22 (10)	Na2^x^—Zn1—Na3^vi^	85.61 (3)
B3—Na1—B2	47.84 (10)	Na3^x^—Zn1—Na3^vi^	129.47 (2)
O1^i^—Na1—B2^iii^	97.93 (10)	O5^iii^—Zn1—Na1^iii^	71.71 (8)
O9^ii^—Na1—B2^iii^	124.59 (10)	O9^vi^—Zn1—Na1^iii^	39.91 (7)
O4—Na1—B2^iii^	144.44 (10)	O10—Zn1—Na1^iii^	127.64 (9)
O7—Na1—B2^iii^	88.40 (10)	O1^ix^—Zn1—Na1^iii^	134.71 (8)
O3^iii^—Na1—B2^iii^	26.37 (8)	Na2^x^—Zn1—Na1^iii^	117.39 (3)
B4^ii^—Na1—B2^iii^	113.11 (11)	Na3^x^—Zn1—Na1^iii^	160.39 (3)
B1^i^—Na1—B2^iii^	107.72 (11)	Na3^vi^—Zn1—Na1^iii^	62.20 (4)
B3—Na1—B2^iii^	116.48 (11)	O5^iii^—Zn1—Na2	83.43 (8)
B2—Na1—B2^iii^	156.47 (6)	O9^vi^—Zn1—Na2	79.97 (7)
O1^i^—Na1—Na3^iv^	45.83 (7)	O10—Zn1—Na2	48.75 (9)
O9^ii^—Na1—Na3^iv^	140.76 (8)	O1^ix^—Zn1—Na2	145.37 (8)
O4—Na1—Na3^iv^	131.83 (8)	Na2^x^—Zn1—Na2	70.674 (14)
O7—Na1—Na3^iv^	99.41 (7)	Na3^x^—Zn1—Na2	103.02 (3)
O3^iii^—Na1—Na3^iv^	52.51 (6)	Na3^vi^—Zn1—Na2	118.00 (3)
B4^ii^—Na1—Na3^iv^	155.89 (9)	Na1^iii^—Zn1—Na2	79.33 (3)
B1^i^—Na1—Na3^iv^	60.76 (8)	O1—B1—O2	121.5 (3)
B3—Na1—Na3^iv^	119.19 (9)	O1—B1—O3	120.0 (3)
B2—Na1—Na3^iv^	115.38 (8)	O2—B1—O3	118.4 (3)
B2^iii^—Na1—Na3^iv^	52.17 (7)	O1—B1—Cd1^xi^	42.59 (16)
O1^i^—Na1—Cd1^v^	90.77 (8)	O2—B1—Cd1^xi^	78.9 (2)
O9^ii^—Na1—Cd1^v^	31.99 (6)	O3—B1—Cd1^xi^	162.6 (2)
O4—Na1—Cd1^v^	56.75 (6)	O1—B1—Na1^xii^	46.55 (16)
O7—Na1—Cd1^v^	112.43 (7)	O2—B1—Na1^xii^	155.6 (3)
O3^iii^—Na1—Cd1^v^	129.97 (8)	O3—B1—Na1^xii^	78.18 (18)
B4^ii^—Na1—Cd1^v^	50.97 (8)	Cd1^xi^—B1—Na1^xii^	85.80 (11)
B1^i^—Na1—Cd1^v^	72.05 (8)	O5—B2—O4	123.4 (3)
B3—Na1—Cd1^v^	84.23 (8)	O5—B2—O3	117.4 (3)
B2—Na1—Cd1^v^	49.21 (8)	O4—B2—O3	119.1 (3)
B2^iii^—Na1—Cd1^v^	154.29 (9)	O5—B2—Na3^xiii^	49.29 (16)
Na3^iv^—Na1—Cd1^v^	132.69 (5)	O4—B2—Na3^xiii^	163.5 (3)
O2^vi^—Na2—O6^iii^	97.04 (9)	O3—B2—Na3^xiii^	71.11 (17)
O2^vi^—Na2—O10^ii^	91.24 (9)	O5—B2—Na1	94.0 (2)
O6^iii^—Na2—O10^ii^	72.03 (10)	O4—B2—Na1	50.05 (18)
O2^vi^—Na2—O6^vi^	58.16 (8)	O3—B2—Na1	130.0 (2)
O6^iii^—Na2—O6^vi^	107.46 (9)	Na3^xiii^—B2—Na1	113.46 (13)
O10^ii^—Na2—O6^vi^	149.35 (10)	O5—B2—Na1^v^	81.3 (2)
O2^vi^—Na2—O4^iii^	131.20 (9)	O4—B2—Na1^v^	127.3 (2)
O6^iii^—Na2—O4^iii^	56.51 (8)	O3—B2—Na1^v^	58.45 (19)
O10^ii^—Na2—O4^iii^	113.25 (11)	Na3^xiii^—B2—Na1^v^	68.70 (10)
O6^vi^—Na2—O4^iii^	89.07 (8)	Na1—B2—Na1^v^	171.50 (14)
O2^vi^—Na2—O10	105.86 (10)	O4—B3—O2	110.6 (3)
O6^iii^—Na2—O10	143.33 (9)	O4—B3—O7	109.8 (3)
O10^ii^—Na2—O10	134.11 (12)	O2—B3—O7	109.4 (3)
O6^vi^—Na2—O10	64.21 (8)	O4—B3—O6	107.9 (3)
O4^iii^—Na2—O10	87.01 (8)	O2—B3—O6	108.8 (3)
O2^vi^—Na2—O8	92.59 (8)	O7—B3—O6	110.4 (3)
O6^iii^—Na2—O8	158.52 (10)	O4—B3—Na2^xiv^	125.4 (2)
O10^ii^—Na2—O8	88.67 (10)	O2—B3—Na2^xiv^	50.56 (15)
O6^vi^—Na2—O8	93.94 (9)	O7—B3—Na2^xiv^	124.7 (2)
O4^iii^—Na2—O8	127.59 (8)	O6—B3—Na2^xiv^	58.25 (17)
O10—Na2—O8	49.20 (7)	O4—B3—Na2^v^	59.21 (16)
O2^vi^—Na2—B5	112.63 (11)	O2—B3—Na2^v^	115.0 (2)
O6^iii^—Na2—B5	150.29 (11)	O7—B3—Na2^v^	135.3 (2)
O10^ii^—Na2—B5	107.34 (12)	O6—B3—Na2^v^	49.90 (16)
O6^vi^—Na2—B5	88.01 (11)	Na2^xiv^—B3—Na2^v^	82.25 (9)
O4^iii^—Na2—B5	99.84 (10)	O4—B3—Na1	52.99 (16)
O10—Na2—B5	26.77 (10)	O2—B3—Na1	129.7 (2)
O8—Na2—B5	28.35 (9)	O7—B3—Na1	56.95 (17)
O2^vi^—Na2—B3^vi^	28.77 (9)	O6—B3—Na1	121.4 (2)
O6^iii^—Na2—B3^vi^	103.60 (10)	Na2^xiv^—B3—Na1	178.31 (15)
O10^ii^—Na2—B3^vi^	119.98 (10)	Na2^v^—B3—Na1	96.30 (12)
O6^vi^—Na2—B3^vi^	29.39 (8)	O9—B4—O6	123.6 (3)
O4^iii^—Na2—B3^vi^	111.57 (10)	O9—B4—O8	118.8 (3)
O10—Na2—B3^vi^	85.15 (10)	O6—B4—O8	117.6 (3)
O8—Na2—B3^vi^	94.07 (10)	O9—B4—Na1^x^	51.91 (17)
B5—Na2—B3^vi^	102.00 (12)	O6—B4—Na1^x^	142.0 (3)
O2^vi^—Na2—B3^iii^	119.08 (10)	O8—B4—Na1^x^	79.6 (2)
O6^iii^—Na2—B3^iii^	28.21 (9)	O10—B5—O7	123.2 (3)
O10^ii^—Na2—B3^iii^	90.05 (11)	O10—B5—O8	118.1 (3)
O6^vi^—Na2—B3^iii^	102.95 (10)	O7—B5—O8	118.7 (3)
O4^iii^—Na2—B3^iii^	28.77 (8)	O10—B5—Na3^x^	77.1 (2)
O10—Na2—B3^iii^	115.71 (10)	O7—B5—Na3^x^	58.31 (18)
O8—Na2—B3^iii^	148.33 (10)	O8—B5—Na3^x^	143.4 (3)
B5—Na2—B3^iii^	124.77 (12)	O10—B5—Na2	69.8 (2)
B3^vi^—Na2—B3^iii^	113.74 (13)	O7—B5—Na2	124.7 (3)
O2^vi^—Na2—Zn1^ii^	58.06 (6)	O8—B5—Na2	75.0 (2)
O6^iii^—Na2—Zn1^ii^	64.38 (6)	Na3^x^—B5—Na2	139.48 (16)
O10^ii^—Na2—Zn1^ii^	37.41 (6)	B1—O1—Cd1^xi^	110.6 (2)
O6^vi^—Na2—Zn1^ii^	113.47 (7)	B1—O1—Zn1^xi^	110.6 (2)
O4^iii^—Na2—Zn1^ii^	120.70 (7)	Cd1^xi^—O1—Zn1^xi^	0.00 (2)
O10—Na2—Zn1^ii^	152.26 (7)	B1—O1—Na1^xii^	108.6 (2)
O8—Na2—Zn1^ii^	105.74 (6)	Cd1^xi^—O1—Na1^xii^	132.05 (12)
B5—Na2—Zn1^ii^	132.99 (9)	Zn1^xi^—O1—Na1^xii^	132.05 (12)
B3^vi^—Na2—Zn1^ii^	85.32 (8)	B1—O1—Na3^xiv^	116.3 (2)
B3^iii^—Na2—Zn1^ii^	91.96 (8)	Cd1^xi^—O1—Na3^xiv^	94.70 (10)
O2^vi^—Na2—Cd1^ii^	58.06 (6)	Zn1^xi^—O1—Na3^xiv^	94.70 (10)
O6^iii^—Na2—Cd1^ii^	64.38 (6)	Na1^xii^—O1—Na3^xiv^	91.77 (10)
O10^ii^—Na2—Cd1^ii^	37.41 (6)	B1—O2—B3	124.8 (3)
O6^vi^—Na2—Cd1^ii^	113.47 (7)	B1—O2—Na2^xiv^	116.0 (2)
O4^iii^—Na2—Cd1^ii^	120.70 (7)	B3—O2—Na2^xiv^	100.66 (18)
O10—Na2—Cd1^ii^	152.26 (7)	B1—O2—Na3^x^	102.4 (2)
O8—Na2—Cd1^ii^	105.74 (6)	B3—O2—Na3^x^	88.90 (18)
B5—Na2—Cd1^ii^	132.99 (9)	Na2^xiv^—O2—Na3^x^	122.91 (10)
B3^vi^—Na2—Cd1^ii^	85.32 (8)	B1—O3—B2	120.8 (3)
B3^iii^—Na2—Cd1^ii^	91.96 (8)	B1—O3—Na1^v^	116.1 (2)
Zn1^ii^—Na2—Cd1^ii^	0.000 (14)	B2—O3—Na1^v^	95.2 (2)
O5^vii^—Na3—O9	115.10 (10)	B1—O3—Na3^xiii^	151.4 (2)
O5^vii^—Na3—O7^ii^	103.02 (10)	B2—O3—Na3^xiii^	80.35 (18)
O9—Na3—O7^ii^	105.90 (9)	Na1^v^—O3—Na3^xiii^	77.25 (7)
O5^vii^—Na3—O1^vi^	104.47 (10)	B2—O4—B3	124.8 (3)
O9—Na3—O1^vi^	114.00 (10)	B2—O4—Na1	104.9 (2)
O7^ii^—Na3—O1^vi^	114.05 (9)	B3—O4—Na1	98.91 (19)
O5^vii^—Na3—O3^vii^	53.80 (8)	B2—O4—Na2^v^	110.9 (2)
O9—Na3—O3^vii^	78.04 (8)	B3—O4—Na2^v^	92.02 (17)
O7^ii^—Na3—O3^vii^	153.61 (9)	Na1—O4—Na2^v^	126.59 (10)
O1^vi^—Na3—O3^vii^	86.56 (8)	B2—O5—Cd1^v^	115.8 (2)
O5^vii^—Na3—B5^ii^	130.19 (12)	B2—O5—Zn1^v^	115.8 (2)
O9—Na3—B5^ii^	97.75 (11)	Cd1^v^—O5—Zn1^v^	0.00 (2)
O7^ii^—Na3—B5^ii^	28.97 (10)	B2—O5—Na3^xiii^	104.5 (2)
O1^vi^—Na3—B5^ii^	93.86 (11)	Cd1^v^—O5—Na3^xiii^	139.36 (13)
O3^vii^—Na3—B5^ii^	175.52 (12)	Zn1^v^—O5—Na3^xiii^	139.36 (13)
O5^vii^—Na3—O10^ii^	138.30 (11)	B4—O6—B3	126.3 (3)
O9—Na3—O10^ii^	104.76 (9)	B4—O6—Na2^v^	117.1 (2)
O7^ii^—Na3—O10^ii^	52.28 (8)	B3—O6—Na2^v^	101.9 (2)
O1^vi^—Na3—O10^ii^	67.74 (8)	B4—O6—Na2^xiv^	108.2 (2)
O3^vii^—Na3—O10^ii^	153.17 (9)	B3—O6—Na2^xiv^	92.4 (2)
B5^ii^—Na3—O10^ii^	26.34 (9)	Na2^v^—O6—Na2^xiv^	107.36 (9)
O5^vii^—Na3—B2^vii^	26.19 (10)	B5—O7—B3	122.4 (3)
O9—Na3—B2^vii^	94.07 (11)	B5—O7—Na3^x^	92.7 (2)
O7^ii^—Na3—B2^vii^	126.07 (11)	B3—O7—Na3^x^	109.2 (2)
O1^vi^—Na3—B2^vii^	101.45 (11)	B5—O7—Na1	122.1 (2)
O3^vii^—Na3—B2^vii^	28.54 (9)	B3—O7—Na1	94.7 (2)
B5^ii^—Na3—B2^vii^	154.88 (13)	Na3^x^—O7—Na1	116.90 (9)
O10^ii^—Na3—B2^vii^	160.82 (11)	B4—O8—B5	121.3 (3)
O5^vii^—Na3—O2^ii^	86.75 (9)	B4—O8—Na2	134.1 (2)
O9—Na3—O2^ii^	68.73 (8)	B5—O8—Na2	76.6 (2)
O7^ii^—Na3—O2^ii^	52.31 (8)	B4—O9—Cd1^xiv^	117.9 (2)
O1^vi^—Na3—O2^ii^	164.75 (9)	B4—O9—Zn1^xiv^	117.9 (2)
O3^vii^—Na3—O2^ii^	108.55 (8)	Cd1^xiv^—O9—Zn1^xiv^	0.00 (2)
B5^ii^—Na3—O2^ii^	70.91 (10)	B4—O9—Na3	117.2 (2)
O10^ii^—Na3—O2^ii^	97.01 (8)	Cd1^xiv^—O9—Na3	108.72 (10)
B2^vii^—Na3—O2^ii^	93.16 (11)	Zn1^xiv^—O9—Na3	108.72 (10)
O5^vii^—Na3—Cd1^ii^	112.99 (8)	B4—O9—Na1^x^	102.1 (2)
O9—Na3—Cd1^ii^	129.54 (8)	Cd1^xiv^—O9—Na1^x^	108.10 (11)
O7^ii^—Na3—Cd1^ii^	76.84 (7)	Zn1^xiv^—O9—Na1^x^	108.10 (11)
O1^vi^—Na3—Cd1^ii^	37.25 (6)	Na3—O9—Na1^x^	100.64 (9)
O3^vii^—Na3—Cd1^ii^	121.39 (7)	B5—O10—Zn1	139.4 (3)
B5^ii^—Na3—Cd1^ii^	60.18 (9)	B5—O10—Na2^x^	123.5 (2)
O10^ii^—Na3—Cd1^ii^	37.07 (5)	Zn1—O10—Na2^x^	96.10 (10)
B2^vii^—Na3—Cd1^ii^	125.68 (9)	B5—O10—Na2	83.5 (2)
O2^ii^—Na3—Cd1^ii^	128.80 (7)	Zn1—O10—Na2	97.68 (11)
O5^vii^—Na3—Zn1^ii^	112.99 (8)	Na2^x^—O10—Na2	102.02 (10)
O9—Na3—Zn1^ii^	129.54 (8)	B5—O10—Na3^x^	76.6 (2)
O7^ii^—Na3—Zn1^ii^	76.84 (7)	Zn1—O10—Na3^x^	83.12 (10)
O1^vi^—Na3—Zn1^ii^	37.25 (6)	Na2^x^—O10—Na3^x^	108.52 (12)
O3^vii^—Na3—Zn1^ii^	121.39 (7)	Na2—O10—Na3^x^	149.21 (11)
B5^ii^—Na3—Zn1^ii^	60.18 (9)		

Symmetry codes: (i) *x*+1/2, −*y*+1/2, −*z*; (ii) *x*+1/2, *y*, −*z*+1/2; (iii) −*x*+3/2, *y*+1/2, *z*; (iv) −*x*+3/2, −*y*+1, *z*−1/2; (v) −*x*+3/2, *y*−1/2, *z*; (vi) −*x*+1, *y*+1/2, −*z*+1/2; (vii) *x*, −*y*+1/2, *z*+1/2; (viii) −*x*+3/2, −*y*+1, *z*+1/2; (ix) −*x*+1/2, *y*+1/2, *z*; (x) *x*−1/2, *y*, −*z*+1/2; (xi) −*x*+1/2, *y*−1/2, *z*; (xii) *x*−1/2, −*y*+1/2, −*z*; (xiii) *x*, −*y*+1/2, *z*−1/2; (xiv) −*x*+1, *y*−1/2, −*z*+1/2.

### Trisodium zinc magnesium pentaborate (II) Crystal data

**Table:**

Na_3_Zn_0.845_Mg_0.155_B_5_O_10_	*D*_x_ = 2.597 Mg m^−^^3^
*M**_r_* = 342.03	Mo *K*α radiation, λ = 0.71073 Å
Orthorhombic, *P**b**c**a*	Cell parameters from 25 reflections
*a* = 7.8931 (12) Å	θ = 10.2–22.2°
*b* = 12.2555 (12) Å	µ = 2.60 mm^−^^1^
*c* = 18.0874 (11) Å	*T* = 293 K
*V* = 1749.7 (3) Å^3^	Prism, colorless
*Z* = 8	0.30 × 0.20 × 0.20 mm
*F*(000) = 1321.7	

### Trisodium zinc magnesium pentaborate (II) Data collection

**Table:**

Rigaku AFC-7R diffractometer	*R*_int_ = 0.053
Radiation source: fine-focus sealed tube	θ_max_ = 30.0°, θ_min_ = 2.3°
2θ – ω scans	*h* = 0→11
Absorption correction: ψ scan (Kopfmann & Huber, 1968)	*k* = 0→17
*T*_min_ = 0.532, *T*_max_ = 0.603	*l* = 0→25
2982 measured reflections	3 standard reflections every 150 reflections
2542 independent reflections	intensity decay: 1.8%
1496 reflections with *I* > 2σ(*I*)	

### Trisodium zinc magnesium pentaborate (II) Refinement

**Table:**

Refinement on *F*^2^	173 parameters
Least-squares matrix: full	0 restraints
*R*[*F*^2^ > 2σ(*F*^2^)] = 0.044	*w* = 1/[σ^2^(*F*_o_^2^) + (0.0554*P*)^2^] where *P* = (*F*_o_^2^ + 2*F*_c_^2^)/3
*wR*(*F*^2^) = 0.113	(Δ/σ)_max_ < 0.001
*S* = 0.87	Δρ_max_ = 1.59 e Å^−^^3^
2542 reflections	Δρ_min_ = −0.70 e Å^−^^3^

### Trisodium zinc magnesium pentaborate (II) Special details

**Table:**

Geometry. All esds (except the esd in the dihedral angle between two l.s. planes) are estimated using the full covariance matrix. The cell esds are taken into account individually in the estimation of esds in distances, angles and torsion angles; correlations between esds in cell parameters are only used when they are defined by crystal symmetry. An approximate (isotropic) treatment of cell esds is used for estimating esds involving l.s. planes.

### Trisodium zinc magnesium pentaborate (II) Fractional atomic coordinates and isotropic or equivalent isotropic displacement parameters (Å^2^)

**Table:**

	*x*	*y*	*z*	*U*_iso_*/*U*_eq_	Occ. (<1)
Na1	0.8316 (2)	0.41813 (15)	0.07839 (9)	0.0239 (4)	
Na2	0.6897 (2)	0.65191 (14)	0.25456 (9)	0.0206 (4)	
Na3	0.7931 (2)	0.42713 (14)	0.42996 (9)	0.0200 (4)	
Zn1	0.42299 (6)	0.67666 (4)	0.09703 (3)	0.01104 (14)	0.845 (5)
Mg1	0.42299 (6)	0.67666 (4)	0.09703 (3)	0.01104 (14)	0.155 (5)
B1	0.4227 (6)	0.1568 (3)	0.0748 (2)	0.0130 (8)	
B2	0.7313 (6)	0.1729 (4)	0.0738 (2)	0.0152 (9)	
B3	0.5641 (6)	0.2867 (4)	0.1632 (2)	0.0125 (8)	
B4	0.5572 (5)	0.3497 (4)	0.2970 (2)	0.0128 (8)	
B5	0.4890 (6)	0.4793 (4)	0.1982 (2)	0.0159 (9)	
O1	0.2775 (3)	0.1191 (2)	0.04430 (15)	0.0143 (6)	
O2	0.4175 (3)	0.2267 (2)	0.13508 (15)	0.0159 (6)	
O3	0.5784 (4)	0.1245 (2)	0.04716 (15)	0.0174 (6)	
O4	0.7209 (4)	0.2457 (2)	0.13007 (15)	0.0180 (6)	
O5	0.8743 (4)	0.1431 (3)	0.04167 (16)	0.0209 (7)	
O6	0.5735 (4)	0.2706 (2)	0.24378 (14)	0.0158 (6)	
O7	0.5439 (4)	0.4035 (2)	0.14602 (14)	0.0168 (6)	
O8	0.5099 (5)	0.4543 (2)	0.27360 (15)	0.0283 (8)	
O9	0.5753 (3)	0.3304 (2)	0.36922 (13)	0.0140 (5)	
O10	0.4240 (4)	0.5735 (2)	0.17972 (16)	0.0247 (7)	

### Trisodium zinc magnesium pentaborate (II) Atomic displacement parameters (Å^2^)

**Table:**

	*U*^11^	*U*^22^	*U*^33^	*U*^12^	*U*^13^	*U*^23^
Na1	0.0291 (10)	0.0218 (9)	0.0206 (9)	−0.0003 (8)	−0.0034 (7)	0.0017 (7)
Na2	0.0211 (9)	0.0221 (8)	0.0186 (8)	−0.0020 (7)	0.0004 (7)	−0.0022 (7)
Na3	0.0235 (9)	0.0211 (9)	0.0154 (7)	0.0011 (8)	−0.0026 (7)	0.0019 (6)
Zn1	0.0112 (2)	0.0106 (2)	0.0112 (2)	−0.0005 (2)	0.0001 (2)	−0.00064 (19)
Mg1	0.0112 (2)	0.0106 (2)	0.0112 (2)	−0.0005 (2)	0.0001 (2)	−0.00064 (19)
B1	0.0127 (18)	0.015 (2)	0.0114 (17)	0.0013 (18)	0.0066 (17)	−0.0031 (14)
B2	0.019 (2)	0.012 (2)	0.0146 (18)	0.0023 (18)	0.0120 (17)	−0.0025 (17)
B3	0.017 (2)	0.0116 (17)	0.0086 (17)	0.0032 (17)	−0.0014 (17)	0.0005 (14)
B4	0.0098 (19)	0.0127 (19)	0.016 (2)	0.0025 (16)	−0.0006 (16)	−0.0045 (15)
B5	0.024 (2)	0.012 (2)	0.011 (2)	0.0034 (18)	−0.0028 (18)	−0.0025 (16)
O1	0.0121 (13)	0.0150 (14)	0.0159 (13)	−0.0008 (11)	0.0042 (11)	−0.0058 (11)
O2	0.0134 (13)	0.0197 (14)	0.0147 (13)	−0.0020 (12)	−0.0010 (12)	−0.0088 (11)
O3	0.0114 (12)	0.0239 (15)	0.0170 (13)	0.0023 (13)	0.0021 (12)	−0.0112 (11)
O4	0.0150 (14)	0.0187 (14)	0.0204 (14)	−0.0008 (12)	−0.0003 (12)	−0.0088 (12)
O5	0.0128 (13)	0.0301 (16)	0.0197 (15)	0.0046 (12)	0.0005 (12)	−0.0089 (13)
O6	0.0278 (15)	0.0098 (12)	0.0100 (12)	0.0043 (12)	−0.0002 (12)	−0.0011 (10)
O7	0.0285 (17)	0.0107 (13)	0.0112 (12)	0.0022 (11)	−0.0014 (11)	−0.0003 (10)
O8	0.058 (2)	0.0135 (14)	0.0133 (14)	0.0107 (15)	−0.0080 (15)	−0.0036 (12)
O9	0.0204 (13)	0.0116 (13)	0.0101 (12)	−0.0002 (12)	−0.0023 (11)	0.0004 (10)
O10	0.0411 (19)	0.0148 (13)	0.0181 (14)	0.0109 (15)	0.0040 (15)	0.0050 (11)

### Trisodium zinc magnesium pentaborate (II) Geometric parameters (Å, º)

**Table:**

Na1—O1^i^	2.305 (3)	B1—O3	1.384 (5)
Na1—O9^ii^	2.399 (3)	B1—O2	1.388 (5)
Na1—O4	2.470 (3)	B1—Mg1^xi^	2.769 (5)
Na1—O7	2.586 (3)	B1—Na1^xii^	3.006 (5)
Na1—O3^iii^	2.687 (3)	B2—O5	1.321 (5)
Na1—B4^ii^	2.992 (5)	B2—O4	1.357 (5)
Na1—B1^i^	3.006 (5)	B2—O3	1.428 (5)
Na1—B3	3.067 (5)	B2—Mg1^v^	2.762 (5)
Na1—B2	3.110 (5)	B2—Na3^xiii^	2.916 (4)
Na1—B2^iii^	3.162 (5)	B2—Na1^v^	3.162 (5)
Na1—Na3^iv^	3.431 (2)	B3—O2	1.462 (5)
Na1—Zn1^v^	3.5530 (18)	B3—O4	1.464 (5)
Na2—O2^vi^	2.354 (3)	B3—O6	1.473 (5)
Na2—O6^iii^	2.377 (3)	B3—O7	1.474 (5)
Na2—O10^ii^	2.399 (4)	B3—Na2^xiv^	2.992 (5)
Na2—O6^vi^	2.536 (3)	B3—Na2^v^	3.039 (5)
Na2—O4^iii^	2.625 (3)	B4—O9	1.335 (5)
Na2—O10	2.675 (4)	B4—O6	1.372 (5)
Na2—O8	2.828 (4)	B4—O8	1.400 (5)
Na2—B5	2.832 (5)	B4—Mg1^xiv^	2.863 (5)
Na2—B3^vi^	2.992 (5)	B4—Na1^x^	2.992 (5)
Na2—B3^iii^	3.039 (5)	B5—O10	1.307 (5)
Na2—Zn1^ii^	3.2693 (17)	B5—O7	1.393 (5)
Na2—Mg1^ii^	3.2693 (17)	B5—O8	1.407 (5)
Na3—O5^vii^	2.288 (3)	B5—Na3^x^	2.859 (5)
Na3—O9	2.360 (3)	O1—Mg1^xi^	1.978 (3)
Na3—O7^ii^	2.427 (3)	O1—Zn1^xi^	1.978 (3)
Na3—O1^vi^	2.462 (3)	O1—Na1^xii^	2.305 (3)
Na3—O3^vii^	2.787 (3)	O1—Na3^xiv^	2.462 (3)
Na3—B5^ii^	2.859 (5)	O2—Na2^xiv^	2.354 (3)
Na3—O10^ii^	2.867 (4)	O2—Na3^x^	2.895 (3)
Na3—O2^ii^	2.895 (3)	O3—Na1^v^	2.687 (3)
Na3—B2^vii^	2.916 (4)	O3—Na3^xiii^	2.787 (3)
Na3—Mg1^ii^	3.2620 (18)	O4—Na2^v^	2.625 (3)
Na3—Zn1^ii^	3.2620 (18)	O5—Mg1^v^	1.932 (3)
Na3—Na1^viii^	3.431 (2)	O5—Zn1^v^	1.932 (3)
Zn1—O5^iii^	1.932 (3)	O5—Na3^xiii^	2.288 (3)
Zn1—O10	1.958 (3)	O6—Na2^v^	2.377 (3)
Zn1—O1^ix^	1.978 (3)	O6—Na2^xiv^	2.536 (3)
Zn1—O9^vi^	1.980 (3)	O7—Na3^x^	2.427 (3)
Zn1—Na3^x^	3.2620 (18)	O9—Mg1^xiv^	1.980 (3)
Zn1—Na2^x^	3.2692 (17)	O9—Zn1^xiv^	1.980 (3)
Zn1—Na3^vi^	3.5456 (18)	O9—Na1^x^	2.399 (3)
Zn1—Na1^iii^	3.5530 (19)	O10—Na2^x^	2.399 (4)
B1—O1	1.353 (5)	O10—Na3^x^	2.867 (4)
			
O1^i^—Na1—O9^ii^	116.11 (11)	O9—Na3—Na1^viii^	115.65 (9)
O1^i^—Na1—O4	97.45 (11)	O7^ii^—Na3—Na1^viii^	137.98 (10)
O9^ii^—Na1—O4	75.55 (10)	O1^vi^—Na3—Na1^viii^	42.18 (7)
O1^i^—Na1—O7	106.21 (11)	O3^vii^—Na3—Na1^viii^	49.89 (7)
O9^ii^—Na1—O7	119.10 (11)	B5^ii^—Na3—Na1^viii^	131.76 (11)
O4—Na1—O7	56.72 (10)	O10^ii^—Na3—Na1^viii^	107.39 (8)
O1^i^—Na1—O3^iii^	91.88 (10)	O2^ii^—Na3—Na1^viii^	152.15 (8)
O9^ii^—Na1—O3^iii^	107.04 (11)	B2^vii^—Na3—Na1^viii^	59.09 (10)
O4—Na1—O3^iii^	168.01 (11)	Mg1^ii^—Na3—Na1^viii^	71.89 (5)
O7—Na1—O3^iii^	113.37 (11)	Zn1^ii^—Na3—Na1^viii^	71.89 (5)
O1^i^—Na1—B4^ii^	141.23 (13)	O5^iii^—Zn1—O10	104.78 (13)
O9^ii^—Na1—B4^ii^	25.79 (10)	O5^iii^—Zn1—O1^ix^	109.67 (11)
O4—Na1—B4^ii^	71.67 (11)	O10—Zn1—O1^ix^	98.11 (13)
O7—Na1—B4^ii^	98.45 (11)	O5^iii^—Zn1—O9^vi^	110.90 (12)
O3^iii^—Na1—B4^ii^	105.36 (12)	O10—Zn1—O9^vi^	112.24 (11)
O1^i^—Na1—B1^i^	25.40 (11)	O1^ix^—Zn1—O9^vi^	119.58 (11)
O9^ii^—Na1—B1^i^	92.03 (12)	O5^iii^—Zn1—Na3^x^	89.02 (10)
O4—Na1—B1^i^	99.92 (12)	O10—Zn1—Na3^x^	60.69 (10)
O7—Na1—B1^i^	128.69 (12)	O1^ix^—Zn1—Na3^x^	48.85 (8)
O3^iii^—Na1—B1^i^	91.74 (11)	O9^vi^—Zn1—Na3^x^	160.08 (9)
B4^ii^—Na1—B1^i^	117.79 (13)	O5^iii^—Zn1—Na2^x^	150.73 (10)
O1^i^—Na1—B3	104.49 (12)	O10—Zn1—Na2^x^	46.78 (9)
O9^ii^—Na1—B3	96.83 (12)	O1^ix^—Zn1—Na2^x^	84.96 (8)
O4—Na1—B3	28.11 (10)	O9^vi^—Zn1—Na2^x^	80.74 (8)
O7—Na1—B3	28.65 (10)	Na3^x^—Zn1—Na2^x^	81.89 (4)
O3^iii^—Na1—B3	141.40 (12)	O5^iii^—Zn1—Na3^vi^	120.77 (10)
B4^ii^—Na1—B3	83.44 (12)	O10—Zn1—Na3^vi^	131.75 (9)
B1^i^—Na1—B3	117.74 (13)	O1^ix^—Zn1—Na3^vi^	81.81 (8)
O1^i^—Na1—B2	74.68 (11)	O9^vi^—Zn1—Na3^vi^	38.94 (8)
O9^ii^—Na1—B2	77.38 (12)	Na3^x^—Zn1—Na3^vi^	129.78 (2)
O4—Na1—B2	24.94 (10)	Na2^x^—Zn1—Na3^vi^	85.55 (4)
O7—Na1—B2	73.87 (11)	O5^iii^—Zn1—Na1^iii^	71.14 (10)
O3^iii^—Na1—B2	166.29 (12)	O10—Zn1—Na1^iii^	127.44 (10)
B4^ii^—Na1—B2	84.31 (13)	O1^ix^—Zn1—Na1^iii^	133.45 (9)
B1^i^—Na1—B2	74.99 (12)	O9^vi^—Zn1—Na1^iii^	39.93 (9)
B3—Na1—B2	47.96 (11)	Na3^x^—Zn1—Na1^iii^	159.71 (4)
O1^i^—Na1—B2^iii^	98.09 (11)	Na2^x^—Zn1—Na1^iii^	117.54 (4)
O9^ii^—Na1—B2^iii^	125.37 (12)	Na3^vi^—Zn1—Na1^iii^	61.85 (4)
O4—Na1—B2^iii^	143.05 (13)	O5^iii^—Zn1—Na2	84.66 (9)
O7—Na1—B2^iii^	86.73 (12)	O10—Zn1—Na2	47.97 (10)
O3^iii^—Na1—B2^iii^	26.71 (10)	O1^ix^—Zn1—Na2	146.06 (9)
B4^ii^—Na1—B2^iii^	112.96 (12)	O9^vi^—Zn1—Na2	80.21 (8)
B1^i^—Na1—B2^iii^	108.33 (13)	Na3^x^—Zn1—Na2	103.06 (4)
B3—Na1—B2^iii^	115.08 (13)	Na2^x^—Zn1—Na2	70.585 (14)
B2—Na1—B2^iii^	156.02 (7)	Na3^vi^—Zn1—Na2	117.98 (4)
O1^i^—Na1—Na3^iv^	45.81 (8)	Na1^iii^—Zn1—Na2	79.88 (4)
O9^ii^—Na1—Na3^iv^	141.90 (10)	O1—B1—O3	120.5 (3)
O4—Na1—Na3^iv^	131.87 (9)	O1—B1—O2	120.4 (4)
O7—Na1—Na3^iv^	99.00 (9)	O3—B1—O2	119.1 (4)
O3^iii^—Na1—Na3^iv^	52.49 (7)	O1—B1—Mg1^xi^	41.80 (17)
B4^ii^—Na1—Na3^iv^	156.20 (11)	O3—B1—Mg1^xi^	162.2 (3)
B1^i^—Na1—Na3^iv^	61.07 (9)	O2—B1—Mg1^xi^	78.6 (2)
B3—Na1—Na3^iv^	118.95 (10)	O1—B1—Na1^xii^	46.95 (18)
B2—Na1—Na3^iv^	116.11 (10)	O3—B1—Na1^xii^	78.0 (2)
B2^iii^—Na1—Na3^iv^	52.31 (8)	O2—B1—Na1^xii^	154.9 (3)
O1^i^—Na1—Zn1^v^	91.58 (8)	Mg1^xi^—B1—Na1^xii^	85.69 (13)
O9^ii^—Na1—Zn1^v^	32.00 (6)	O5—B2—O4	124.3 (4)
O4—Na1—Zn1^v^	56.24 (7)	O5—B2—O3	117.3 (4)
O7—Na1—Zn1^v^	112.11 (8)	O4—B2—O3	118.4 (3)
O3^iii^—Na1—Zn1^v^	131.28 (9)	O5—B2—Mg1^v^	39.4 (2)
B4^ii^—Na1—Zn1^v^	50.99 (9)	O4—B2—Mg1^v^	86.2 (3)
B1^i^—Na1—Zn1^v^	72.70 (9)	O3—B2—Mg1^v^	153.3 (3)
B3—Na1—Zn1^v^	83.71 (9)	O5—B2—Na3^xiii^	49.38 (19)
B2—Na1—Zn1^v^	48.41 (9)	O4—B2—Na3^xiii^	162.9 (3)
B2^iii^—Na1—Zn1^v^	155.55 (10)	O3—B2—Na3^xiii^	70.5 (2)
Na3^iv^—Na1—Zn1^v^	133.71 (6)	Mg1^v^—B2—Na3^xiii^	88.71 (12)
O2^vi^—Na2—O6^iii^	96.54 (11)	O5—B2—Na1	93.5 (3)
O2^vi^—Na2—O10^ii^	90.74 (11)	O4—B2—Na1	50.1 (2)
O6^iii^—Na2—O10^ii^	71.31 (11)	O3—B2—Na1	128.7 (3)
O2^vi^—Na2—O6^vi^	58.14 (9)	Mg1^v^—B2—Na1	74.21 (12)
O6^iii^—Na2—O6^vi^	107.09 (11)	Na3^xiii^—B2—Na1	112.80 (14)
O10^ii^—Na2—O6^vi^	148.77 (12)	O5—B2—Na1^v^	82.7 (3)
O2^vi^—Na2—O4^iii^	130.80 (11)	O4—B2—Na1^v^	128.3 (3)
O6^iii^—Na2—O4^iii^	56.64 (10)	O3—B2—Na1^v^	57.7 (2)
O10^ii^—Na2—O4^iii^	113.16 (12)	Mg1^v^—B2—Na1^v^	99.65 (14)
O6^vi^—Na2—O4^iii^	88.79 (10)	Na3^xiii^—B2—Na1^v^	68.60 (11)
O2^vi^—Na2—O10	106.68 (11)	Na1—B2—Na1^v^	173.51 (18)
O6^iii^—Na2—O10	142.66 (11)	O2—B3—O4	110.8 (3)
O10^ii^—Na2—O10	135.35 (14)	O2—B3—O6	108.5 (3)
O6^vi^—Na2—O10	64.51 (10)	O4—B3—O6	108.5 (3)
O4^iii^—Na2—O10	86.21 (10)	O2—B3—O7	109.2 (3)
O2^vi^—Na2—O8	92.86 (10)	O4—B3—O7	109.8 (3)
O6^iii^—Na2—O8	158.26 (12)	O6—B3—O7	110.1 (3)
O10^ii^—Na2—O8	89.05 (11)	O2—B3—Na2^xiv^	50.63 (17)
O6^vi^—Na2—O8	94.52 (10)	O4—B3—Na2^xiv^	125.5 (3)
O4^iii^—Na2—O8	127.91 (10)	O6—B3—Na2^xiv^	57.85 (19)
O10—Na2—O8	50.26 (9)	O7—B3—Na2^xiv^	124.7 (3)
O2^vi^—Na2—B5	113.27 (13)	O2—B3—Na2^v^	115.0 (2)
O6^iii^—Na2—B5	150.17 (13)	O4—B3—Na2^v^	59.70 (19)
O10^ii^—Na2—B5	108.08 (13)	O6—B3—Na2^v^	49.99 (18)
O6^vi^—Na2—B5	88.55 (12)	O7—B3—Na2^v^	135.4 (3)
O4^iii^—Na2—B5	99.71 (12)	Na2^xiv^—B3—Na2^v^	81.82 (11)
O10—Na2—B5	27.27 (11)	O2—B3—Na1	129.4 (2)
O8—Na2—B5	28.79 (11)	O4—B3—Na1	52.65 (19)
O2^vi^—Na2—B3^vi^	28.69 (10)	O6—B3—Na1	122.0 (3)
O6^iii^—Na2—B3^vi^	103.26 (12)	O7—B3—Na1	57.23 (19)
O10^ii^—Na2—B3^vi^	119.39 (12)	Na2^xiv^—B3—Na1	178.11 (17)
O6^vi^—Na2—B3^vi^	29.45 (10)	Na2^v^—B3—Na1	96.73 (14)
O4^iii^—Na2—B3^vi^	111.38 (12)	O9—B4—O6	123.5 (4)
O10—Na2—B3^vi^	85.69 (12)	O9—B4—O8	119.1 (3)
O8—Na2—B3^vi^	94.37 (12)	O6—B4—O8	117.3 (3)
B5—Na2—B3^vi^	102.52 (14)	O9—B4—Mg1^xiv^	37.62 (17)
O2^vi^—Na2—B3^iii^	118.64 (12)	O6—B4—Mg1^xiv^	86.7 (2)
O6^iii^—Na2—B3^iii^	28.33 (10)	O8—B4—Mg1^xiv^	153.5 (3)
O10^ii^—Na2—B3^iii^	89.67 (13)	O9—B4—Na1^x^	51.42 (19)
O6^vi^—Na2—B3^iii^	102.61 (12)	O6—B4—Na1^x^	141.5 (3)
O4^iii^—Na2—B3^iii^	28.79 (10)	O8—B4—Na1^x^	79.2 (2)
O10—Na2—B3^iii^	114.92 (12)	Mg1^xiv^—B4—Na1^x^	74.69 (11)
O8—Na2—B3^iii^	148.49 (12)	O10—B5—O7	122.5 (4)
B5—Na2—B3^iii^	124.60 (13)	O10—B5—O8	119.1 (4)
B3^vi^—Na2—B3^iii^	113.48 (16)	O7—B5—O8	118.3 (3)
O2^vi^—Na2—Zn1^ii^	58.00 (7)	O10—B5—Na2	69.6 (2)
O6^iii^—Na2—Zn1^ii^	64.38 (7)	O7—B5—Na2	124.6 (3)
O10^ii^—Na2—Zn1^ii^	36.50 (7)	O8—B5—Na2	75.5 (2)
O6^vi^—Na2—Zn1^ii^	113.47 (8)	O10—B5—Na3^x^	77.2 (2)
O4^iii^—Na2—Zn1^ii^	120.82 (8)	O7—B5—Na3^x^	58.0 (2)
O10—Na2—Zn1^ii^	152.94 (9)	O8—B5—Na3^x^	143.1 (3)
O8—Na2—Zn1^ii^	105.19 (8)	Na2—B5—Na3^x^	139.58 (18)
B5—Na2—Zn1^ii^	132.82 (11)	B1—O1—Mg1^xi^	111.1 (2)
B3^vi^—Na2—Zn1^ii^	85.28 (9)	B1—O1—Zn1^xi^	111.1 (2)
B3^iii^—Na2—Zn1^ii^	92.06 (9)	Mg1^xi^—O1—Zn1^xi^	0.00 (3)
O2^vi^—Na2—Mg1^ii^	58.00 (7)	B1—O1—Na1^xii^	107.6 (2)
O6^iii^—Na2—Mg1^ii^	64.38 (7)	Mg1^xi^—O1—Na1^xii^	133.06 (14)
O10^ii^—Na2—Mg1^ii^	36.50 (7)	Zn1^xi^—O1—Na1^xii^	133.06 (14)
O6^vi^—Na2—Mg1^ii^	113.47 (8)	B1—O1—Na3^xiv^	116.2 (2)
O4^iii^—Na2—Mg1^ii^	120.82 (8)	Mg1^xi^—O1—Na3^xiv^	93.93 (11)
O10—Na2—Mg1^ii^	152.94 (9)	Zn1^xi^—O1—Na3^xiv^	93.93 (11)
O8—Na2—Mg1^ii^	105.19 (8)	Na1^xii^—O1—Na3^xiv^	92.00 (10)
B5—Na2—Mg1^ii^	132.82 (11)	B1—O2—B3	124.1 (3)
B3^vi^—Na2—Mg1^ii^	85.28 (9)	B1—O2—Na2^xiv^	115.9 (2)
B3^iii^—Na2—Mg1^ii^	92.06 (9)	B3—O2—Na2^xiv^	100.7 (2)
Zn1^ii^—Na2—Mg1^ii^	0.000 (19)	B1—O2—Na3^x^	102.4 (2)
O5^vii^—Na3—O9	115.24 (12)	B3—O2—Na3^x^	89.0 (2)
O5^vii^—Na3—O7^ii^	103.12 (12)	Na2^xiv^—O2—Na3^x^	123.59 (12)
O9—Na3—O7^ii^	105.71 (11)	B1—O3—B2	120.6 (3)
O5^vii^—Na3—O1^vi^	104.82 (12)	B1—O3—Na1^v^	115.3 (2)
O9—Na3—O1^vi^	113.76 (11)	B2—O3—Na1^v^	95.6 (2)
O7^ii^—Na3—O1^vi^	113.93 (11)	B1—O3—Na3^xiii^	151.6 (2)
O5^vii^—Na3—O3^vii^	54.07 (9)	B2—O3—Na3^xiii^	80.6 (2)
O9—Na3—O3^vii^	78.29 (10)	Na1^v^—O3—Na3^xiii^	77.62 (8)
O7^ii^—Na3—O3^vii^	154.12 (11)	B2—O4—B3	125.7 (3)
O1^vi^—Na3—O3^vii^	86.29 (10)	B2—O4—Na1	104.9 (3)
O5^vii^—Na3—B5^ii^	130.45 (14)	B3—O4—Na1	99.2 (2)
O9—Na3—B5^ii^	97.41 (13)	B2—O4—Na2^v^	109.9 (3)
O7^ii^—Na3—B5^ii^	29.12 (11)	B3—O4—Na2^v^	91.5 (2)
O1^vi^—Na3—B5^ii^	93.56 (12)	Na1—O4—Na2^v^	127.18 (12)
O3^vii^—Na3—B5^ii^	175.18 (13)	B2—O5—Mg1^v^	114.9 (3)
O5^vii^—Na3—O10^ii^	138.21 (12)	B2—O5—Zn1^v^	114.9 (3)
O9—Na3—O10^ii^	104.75 (10)	Mg1^v^—O5—Zn1^v^	0.00 (3)
O7^ii^—Na3—O10^ii^	52.34 (9)	B2—O5—Na3^xiii^	104.6 (3)
O1^vi^—Na3—O10^ii^	67.33 (9)	Mg1^v^—O5—Na3^xiii^	140.35 (15)
O3^vii^—Na3—O10^ii^	152.54 (10)	Zn1^v^—O5—Na3^xiii^	140.35 (15)
B5^ii^—Na3—O10^ii^	26.39 (11)	B4—O6—B3	126.6 (3)
O5^vii^—Na3—O2^ii^	86.84 (11)	B4—O6—Na2^v^	116.6 (2)
O9—Na3—O2^ii^	68.39 (9)	B3—O6—Na2^v^	101.7 (2)
O7^ii^—Na3—O2^ii^	52.57 (9)	B4—O6—Na2^xiv^	108.7 (2)
O1^vi^—Na3—O2^ii^	164.65 (10)	B3—O6—Na2^xiv^	92.7 (2)
O3^vii^—Na3—O2^ii^	108.84 (9)	Na2^v^—O6—Na2^xiv^	106.97 (11)
B5^ii^—Na3—O2^ii^	71.15 (11)	B5—O7—B3	122.6 (3)
O10^ii^—Na3—O2^ii^	97.33 (9)	B5—O7—Na3^x^	92.9 (2)
O5^vii^—Na3—B2^vii^	26.00 (12)	B3—O7—Na3^x^	108.9 (2)
O9—Na3—B2^vii^	94.71 (13)	B5—O7—Na1	123.2 (3)
O7^ii^—Na3—B2^vii^	126.27 (13)	B3—O7—Na1	94.1 (2)
O1^vi^—Na3—B2^vii^	101.24 (12)	Na3^x^—O7—Na1	116.11 (12)
O3^vii^—Na3—B2^vii^	28.88 (11)	B4—O8—B5	121.6 (3)
B5^ii^—Na3—B2^vii^	155.19 (14)	B4—O8—Na2	133.4 (3)
O10^ii^—Na3—B2^vii^	160.11 (13)	B5—O8—Na2	75.8 (2)
O2^ii^—Na3—B2^vii^	93.60 (12)	B4—O9—Mg1^xiv^	118.1 (2)
O5^vii^—Na3—Mg1^ii^	113.38 (10)	B4—O9—Zn1^xiv^	118.1 (2)
O9—Na3—Mg1^ii^	129.07 (9)	Mg1^xiv^—O9—Zn1^xiv^	0.00 (4)
O7^ii^—Na3—Mg1^ii^	76.74 (8)	B4—O9—Na3	116.4 (2)
O1^vi^—Na3—Mg1^ii^	37.22 (6)	Mg1^xiv^—O9—Na3	109.23 (12)
O3^vii^—Na3—Mg1^ii^	121.18 (8)	Zn1^xiv^—O9—Na3	109.23 (12)
B5^ii^—Na3—Mg1^ii^	59.94 (10)	B4—O9—Na1^x^	102.8 (2)
O10^ii^—Na3—Mg1^ii^	36.55 (6)	Mg1^xiv^—O9—Na1^x^	108.08 (12)
O2^ii^—Na3—Mg1^ii^	128.91 (8)	Zn1^xiv^—O9—Na1^x^	108.08 (12)
B2^vii^—Na3—Mg1^ii^	125.45 (11)	Na3—O9—Na1^x^	100.09 (11)
O5^vii^—Na3—Zn1^ii^	113.38 (10)	B5—O10—Zn1	140.1 (3)
O9—Na3—Zn1^ii^	129.07 (9)	B5—O10—Na2^x^	122.0 (3)
O7^ii^—Na3—Zn1^ii^	76.74 (8)	Zn1—O10—Na2^x^	96.72 (12)
O1^vi^—Na3—Zn1^ii^	37.22 (6)	B5—O10—Na2	83.1 (3)
O3^vii^—Na3—Zn1^ii^	121.18 (8)	Zn1—O10—Na2	99.08 (13)
B5^ii^—Na3—Zn1^ii^	59.94 (10)	Na2^x^—O10—Na2	102.12 (11)
O10^ii^—Na3—Zn1^ii^	36.55 (6)	B5—O10—Na3^x^	76.5 (2)
O2^ii^—Na3—Zn1^ii^	128.91 (8)	Zn1—O10—Na3^x^	82.76 (11)
B2^vii^—Na3—Zn1^ii^	125.45 (11)	Na2^x^—O10—Na3^x^	108.41 (13)
Mg1^ii^—Na3—Zn1^ii^	0.000 (17)	Na2—O10—Na3^x^	149.01 (13)
O5^vii^—Na3—Na1^viii^	66.25 (9)		

Symmetry codes: (i) *x*+1/2, −*y*+1/2, −*z*; (ii) *x*+1/2, *y*, −*z*+1/2; (iii) −*x*+3/2, *y*+1/2, *z*; (iv) −*x*+3/2, −*y*+1, *z*−1/2; (v) −*x*+3/2, *y*−1/2, *z*; (vi) −*x*+1, *y*+1/2, −*z*+1/2; (vii) *x*, −*y*+1/2, *z*+1/2; (viii) −*x*+3/2, −*y*+1, *z*+1/2; (ix) −*x*+1/2, *y*+1/2, *z*; (x) *x*−1/2, *y*, −*z*+1/2; (xi) −*x*+1/2, *y*−1/2, *z*; (xii) *x*−1/2, −*y*+1/2, −*z*; (xiii) *x*, −*y*+1/2, *z*−1/2; (xiv) −*x*+1, *y*−1/2, −*z*+1/2.
